# Laboratory Parameters are Possible Prognostic Markers in Patients with Advanced-stage NSCLC Treated with Bevacizumab plus Chemotherapy

**DOI:** 10.7150/jca.58851

**Published:** 2021-07-30

**Authors:** Martin Svaton, Jiri Blazek, Gabriela Krakorova, Marcela Buresova, Zuzana Teufelova, Josef Vodicka, Karolina Hurdalkova, Magda Barinova, Milos Pesek

**Affiliations:** 1 Department of Pneumology and Phthisiology, University hospital in Pilsen, Charles University, Faculty of Medicine in Pilsen, Pilsen, Czech Republic; 2 Department of Surgery, Charles University, Faculty of Medicine in Pilsen, Pilsen, Czech Republic; 3 Institute of Biostatistics and Analyses, Ltd. Brno, Czech Republic

**Keywords:** bevacizumab, inflammation, CRP, albumin, NSCLC, predictive, prognostic

## Abstract

**Purpose:** To investigate potential associations between selected laboratory markers (CRP, LDH, albumin, sodium, hemoglobin, neutrophils, and neutrophils/lymphocytes ratio [NLR]) and outcomes in patients with non-small cell lung cancer (NSCLC) treated with bevacizumab (BEV) plus chemotherapy.

**Patients and Methods:** We retrospectively analyzed 105 patients with NSCLC from the Czech TULUNG registry treated at University Hospital in Pilsen with BEV + chemotherapy. Response to therapy was tested by Fisher's exact test. Survival statistics were evaluated using the Kaplan-Meier method and Cox analysis.

**Results:** We showed significantly better disease control rate when CRP, albumin, hemoglobin, and NLR were within established “normal” values. In univariate analysis, normal values of CRP, LDH, albumin, sodium, hemoglobin, neutrophils, and NLR were associated with better overall survival (OS). Normal values of CRP, albumin, hemoglobin, neutrophils, and NLR were associated also with better progression-free survival (PFS). In a multivariate Cox model, normal values of LDH, albumin, and NLR were associated with significantly better OS while normal CRP, albumin, and NLR were associated with better PFS.

**Conclusions:** LDH and sodium appear to be possible prognostic markers for BEV treatment in combination with chemotherapy in NSCLC. The parameters associated with inflammatory response (CRP, NLR, albumin, and possibly hemoglobin) appear to be promising predictive markers for this treatment combination.

## Introduction

Bevacizumab (BEV) is an intravenously administered monoclonal antibody targeting vascular endothelial growth factor (VEGF) that is widely used in treating patients with advanced non-small cell lung cancer (NSCLC) 1. The Eastern Cooperative Oncology Group (ECOG) 4599 phase III trial showed a significant survival benefit from using BEV in combination with carboplatin and paclitaxel (CP) in comparison with CP chemotherapy alone in patients with previously untreated advanced, metastatic, or recurrent NSCLC 2. Such results were achieved using BEV as maintenance therapy until disease progression. It has been demonstrated previously that the superiority of BEV is limited to patients with non-squamous histology due to higher proportion of potentially risky hemoptysis in squamous lung cancers 3. Aside from the non-squamous histology, there remains to date no molecular biomarker available for predicting treatment efficacy of bevacizumab-based therapy.

A number of past studies have sought an effective predictive marker for this treatment 4-7. In particular, there have been attempts to use the expression of VEGF, the effect of arterial hypertension, or examination by perfusion computed tomography (CT) to determine the effect of angiogenesis in a given tumor 4-6. None of these, however, have been found sufficiently reliable or subsequently verified satisfactorily by prospective work to be put into routine clinical practice 3.

Laboratory parameters have been shown to be potential predictors of treatment in a number of other studies involving NSCLC 8-11. Moreover, some laboratory parameters (mainly C-reactive protein [CRP], lactate dehydrogenase [LDH], and neutrophils/lymphocytes ratio [NLR]) have shown potential as prognostic or even predictive markers of bevacizumab-based therapy in tumors relating to colorectal cancer, breast cancer, pancreatic cancer, renal cell cancer, glioblastoma, and ovarian cancer 12-17. This topic has not been comprehensively studied, however, for the combination of chemotherapy and bevacizumab in NSCLC. These laboratory findings may be important decision-making inputs prior to initiating various immunotherapy options, including that of the chemotherapy + bevacizumab combination providing a therapeutic basis for further combination with atezolizumab.

Given this background, the aim of the present study was to investigate potential associations between selected laboratory markers and outcomes in NSCLC patients treated with BEV plus chemotherapy.

## Patients and Methods

### Study design and treatment

Clinical data of patients with cytologically or histologically confirmed advanced NSCLC treated with BEV and chemotherapy (mainly CP) were analyzed retrospectively. The patients were treated in the first, or rarely in the second, line of treatment at the Department of Pneumology and Phthisiology, University Hospital in Pilsen in the Czech Republic between 2010 and 2020. BEV was administered intravenously at the approved dose of 7.5 mg/kg every 3 weeks together with standard platinum doublet chemotherapy. The BEV treatment was administered until progression or unacceptable toxicity, and chemotherapy was given to 4 cycles. Clinical follow-up including physical examination, chest X-ray, and routine laboratory tests was made at least every 4 weeks. CT or positron-emission tomography (PET)/CT were performed at regular intervals according to the local standards or, when progression was suspected, based on clinical or chest X-ray examination. Laboratory markers investigated in the present study included CRP, LDH, albumin, sodium (Na), calcium (Ca), hemoglobin (Hb), neutrophils (Neu), lymphocytes, and NLR. These markers were measured at the initiation of bevacizumab treatment. Serving as the data source was TULUNG, a national non-interventional post-registration database of epidemiological and clinical data for patients with advanced-stage NSCLC treated with targeted or biological therapies in the Czech Republic. We used data recorded from our center (University Hospital in Pilsen) relating to all of our patients in the register who had been treated with chemotherapy and bevacizumab. The patients had given their informed consent to be included into this database and for use of these data for scientific purposes.

### Statistical methods

Standard frequency tables and descriptive statistics were used to characterize the sample data set. The overall response rate (ORR) was defined as the best response according to the Response Evaluation Criteria in Solid Tumors (RECIST 1.1) 18. We compared disease control rates between selected groups. Continuous parameters are described herein using mean with 95% confidence interval (CI) and median with minimum and maximum, together with the total number of non-missing observations. Categorical parameters were summarized using absolute and relative frequencies. Relative frequencies were calculated based on the number of patients in a relevant subgroup. ORR (difference in complete response + partial response + stable disease between two categories [i.e., parameter within normal range and outside the norm]) was tested by Fisher's exact test. Overall survival (OS) was defined as the time from initiating treatment to the date of death due to any cause. Progression-free survival (PFS) was defined as the time from treatment initiation to the date of first documented progression or death due to any cause. OS and PFS were estimated using the Kaplan-Meier method, and all point estimates include 95% confidence intervals (95% CI). Differences between OS and PFS were tested by log-rank test. Finally, a multivariate Cox proportional hazards model was used to evaluate the relationship of all potential prognostic factors to the survival measures. The cutoff for deciding on statistical significance was set at α=0.05.

The cutoff for laboratory parameters was “normal level” versus “abnormal value” (i.e., >8 mg/l for CRP, >4.2 µkat/l for LDH, <35 g/l for albumin, <137 mmol/l for Na, <2.1 or >2.6 mmol/l for Ca, <135 g/l for men and <120 g/l for women for Hb, >7×10^9^/l for Neu, and <0.8×10^9^/l for lymphocytes). Cutoff values are based on standardized lower / upper limits from our certified hematological and biochemical laboratory. The cutoff for NLR was set at the median of reference NLR values (i.e., 3.3101).

Statistical analyses were performed using IBM SPSS, Statistics (version 25.0), and R software (version 3.5.1).

## Results

### Patient characteristics

Included into the retrospective analysis were 105 patients, consisting of 65 males and 40 females, with median age of 63 years. The baseline patient characteristics are summarized in Table 1. Due to the low numbers of values outside the normal limits for Ca and lymphocytes, these laboratory parameters could not be further included into the calculated analyses.

### Overall response rate (ORR)

We showed a significant relationship between ORR (difference in complete response + partial response + stable disease between two categories) and CRP, albumin, Hb, and NLR, as well as a trend in ORR relative to Neu (p=0.058). Values of these laboratory parameters outside the established standards were associated with poorer disease control rate. All results are summarized in Table 2.

### Univariate analysis of PFS and OS

**Figure 1:**
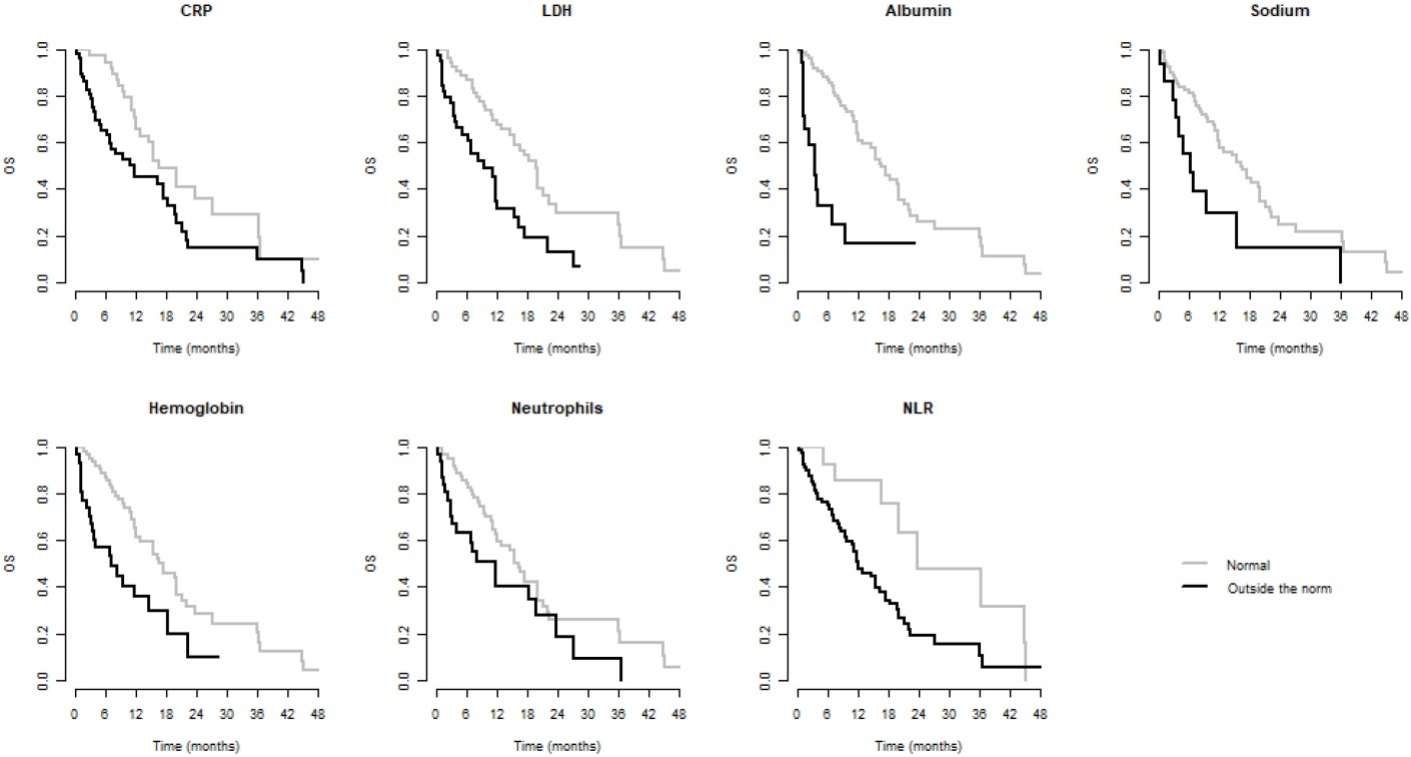
Kaplan-Meier curves of parameters significant for OS

**Figure 2:**
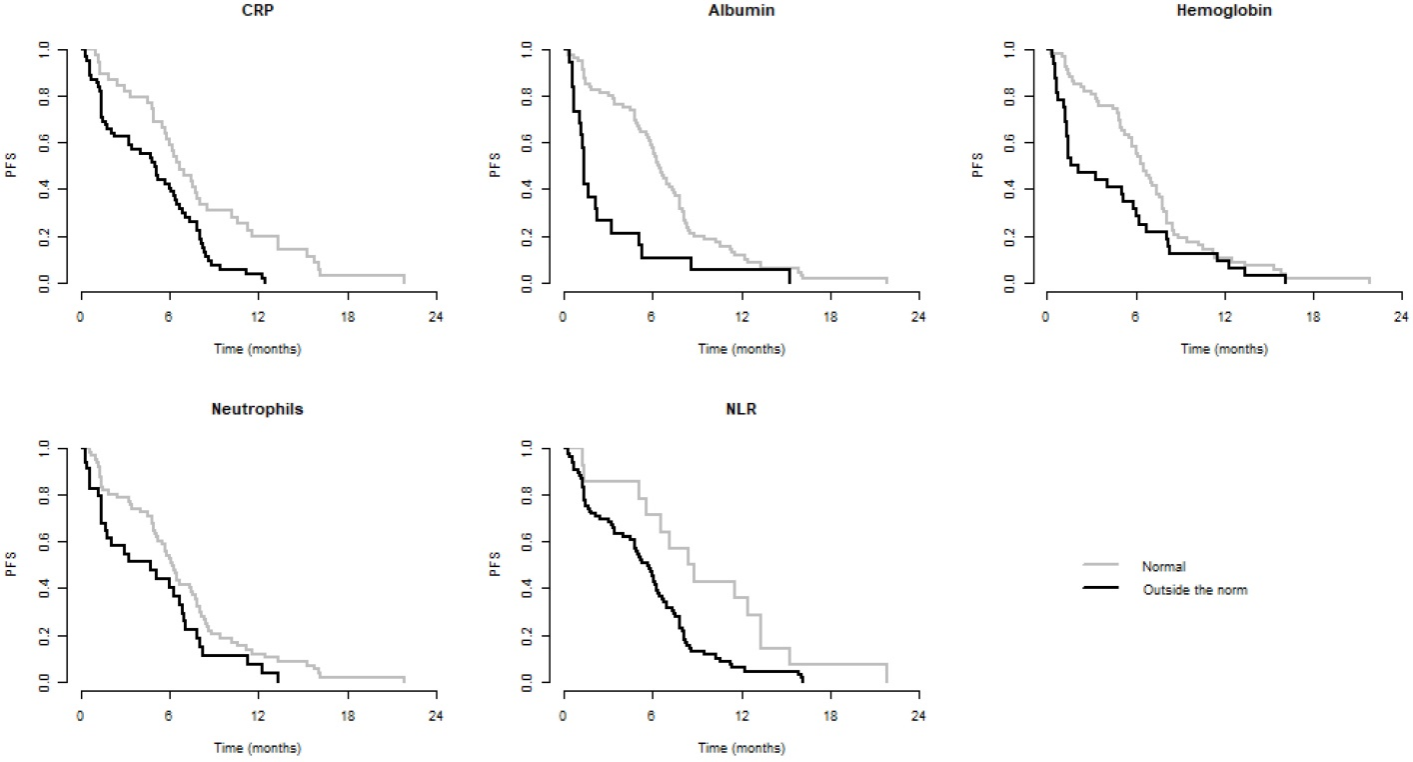
Kaplan-Meier curves of parameters significant for PFS

We observed significantly better OS when CRP, LDH, albumin, Na, Hb, Neu, and NLR were within normal and significantly better PFS when CRP, albumin, Hb, Neu, and NLR were normal. Values of these laboratory parameters outside the established standards were associated with poorer PFS and/or OS. These results are summarized in Table 3. Kaplan-Meier curves for PFS and OS of significant parameters are shown in figures 1 and 2.

### Multivariate Cox proportional hazards model

A Cox model was made for demographic variables (age, gender, smoking status, ECOG performance status [PS]) and for variables determined as significant for predicting OS or PFS in univariate analysis. We observed significantly better OS for within-normal LDH, albumin, and NLR, as well as a trend relative to Na (p=0.077). Values of these laboratory parameters outside the established standards were associated with poorer OS. We observed significantly better PFS for ECOG PS, CRP, albumin, and NLR within normal. Values of CRP, albumin, and NLR outside the established standards were associated with poorer PFS. Results are summarized in Table 4.

## Discussion

The data from the present retrospective analysis indicate possible prognostic and theoretically predictive value for some of those laboratory parameters examined. To the best of our knowledge, this work is the first in the English-language literature (within the PubMed Database) to date to examine the relationships between CRP, Hb, and Na and the efficacy of bevacizumab treatment in NSCLC. Our work is also unique in relation to the complexity of the baseline aspects of laboratory marker values with respect to the effect of bevacizumab treatment in NSCLC.

LDH and Na showed possible prognostic value in NSCLC patients treated with bevacizumab plus chemotherapy. Adverse prognostic effects of hyponatremia on patients with NSCLC have been demonstrated in other studies concerning NSCLC and other treatment combinations 8, 19. We found no other studies dealing with the relationship between Na and bevacizumab treatment in NSCLC. With regard to LDH, the literature is much richer. A meta-analysis of the relationship between LDH and bevacizumab treatment in colorectal cancer (CRC) showed significant relationships for both PFS and OS 20. The effect on the response to treatment has not been proven, however, so a question remains whether the relationship with LDH is predictive or only prognostic in CRC. In our study of NSCLC, we demonstrated only a prognostic value. The prognostic value of LDH in NSCLC patients treated with bevacizumab was demonstrated also by the study of Honag et al. 21. Li et al. then demonstrated a relationship between LDH and both OS and PFS in a univariate model, but this was not confirmed in Cox's multidimensional model 22. On the contrary, they point to the possible importance for the development of LDH levels during treatment with bevacizumab. This was suggested, too, by a study from Silvestis et al. in relation to CRC 23. This can be supported also by preclinical data suggesting a relationship between LDH, angiogenesis, and VEGF 24, 25. Because serum level of LDH was shown to be an indirect factor indicative for hypoxia in tumor tissues with large tumor burden, it seems plausible that the improvement of hypoxia in the tumor microenvironment by bevacizumab could be reflected in a decrease of LDH serum level 22. It was not, however, a goal of our work to examine this. In our opinion, the baseline LDH can only be considered as a prognostic marker. For it to have possible predictive value, it would be necessary to verify the relationship to its changes during BEV treatment. Finally, different LDH isoforms might have different functions in this setting 26, and this is not normally detected in routine laboratory samples. This can complicate the potential for LDH to be used in routine clinical practice.

The parameters associated with the inflammatory tumor microenvironment 10 are, in our view, more promising predictive markers for BEV treatment in NSCLC. VEGF, a target of BEV, is a soluble dimeric protein with multiple bioregulative activities that is mainly released in hypoxic and inflammatory conditions 27. VEGF bioregulative activity is very complex, and it also involves the anticancer immune system 27. Several studies in relation to CRC, breast cancer, and renal cell cancer have demonstrated that bevacizumab is most advantageous in extending PFS and/or OS in patients who presented a lower systemic inflammatory profile prior to beginning the treatment 12, 13, 15, 28-31. These studies proved the positive relationship between unincreased values of Neu or NLR and CRP, as well as of higher Hb values, and better results of BEV treatment 12, 13, 15, 28-31. An effect of inflammation on poorer treatment outcomes has been demonstrated also with other treatment modalities in NSCLC 9-11. We also found two studies directly addressing the relationship of selected parameters associated with inflammatory response to BEV treatment in NSCLC 21, 32. Hoang et al. demonstrate in their study an adverse effect of hypoalbuminemia on OS and PFS 21. Botta et al. then point to a possible effect from high numbers of circulating neutrophils and monocytes as well as high NLR on the results of bevacizumab treatment 32. Neither of these studies, however, addressed the relationship to ORR. They also did not consider the usefulness of CRP and Hb as important parameters of inflammation 10. In this regard, our work is the most comprehensive of its kind in predicting BEV therapy. We demonstrated the relationship between CRP, albumin, Hb, Neu, and NLR on ORR and also that of CRP, albumin, and NLR on PFS in a multivariate Cox model. Only Hb did not confirm its relationship to FPS from the univariate model in the Cox model. Although decline in Hb during ongoing inflammation is known, its decrease may be associated with other effects that may have been reflected in the Cox model 33, 34.

The present study has several limitations. First, this was a retrospective study that could be biased with regard to patient selection for BEV plus chemotherapy regimen. Secondly, PFS was not confirmed by an independent board. Finally, we examined a relatively limited number of patients and therefore some analyses may lack sufficient statistical power. The present report should thus be regarded as exploratory and the results should be verified in a larger, prospective study. Determining the optimal cutoff for NLR (which has varied from 2.5 to 5 in studies analyzing the possible relationship of NLR to the outcome of bevacizumab treatment) may also be problematic for subsequent validation 12, 28, 29, 32, 35, 36.

## Conclusions

LDH and Na appear to be possible prognostic markers for bevacizumab treatment in combination with chemotherapy in NSCLC. The parameters associated with the inflammatory response (CRP, NLR, albumin, and possibly Hb), then, appear to be promising predictive markers for this treatment combination. It would be appropriate to verify their use (probably and preferably in the form of a proinflammatory index) in a prospective study.

## Figures and Tables

**Table 1:** Baseline patient characteristics.

Parameter	Category	n (%)
Sex	Male	65 (61.9%)
	Female	40 (38.1%)
Smoking status	Non-smoker	22 (21.0%)
	Former smoker	31 (29.5%)
	Smoker	52 (49.5%)
ECOG PS	0	4 (3.8%)
	1	93 (88.6%)
	2	8 (7.6%)
Line of therapy	First	104 (99.1%)
	Second	1 (1.0%)
Histology	Adenocarcinoma	100 (95.2%)
	Other	5 (4.8%)
T (TNM classification)	TX	4 (3.8%)
	T1 (valid until 1. 1. 2011)	3 (2.9%)
	T1a	1 (1.0%)
	T1b	8 (7.6%)
	T2 (valid until 1. 1. 2011)	6 (5.7%)
	T2a	11 (10.5%)
	T2b	7 (6.7%)
	T3	19 (18.1%)
	T4	46 (43.8%)
N (TNM classification)	NX	5 (4.8%)
	N0	23 (21.9%)
	N1	9 (8.6%)
	N2	23 (21.9%)
	N3	45 (42.9%)
M (TNM classification)	MX	1 (1.0%)
	M0	12 (11.4%)
	M1 (valid until 1. 1. 2011)	17 (16.2%)
	M1a	27 (25.7%)
	M1b	44 (41.9%)
	M1c (valid from TNM8)	4 (3.8%)
Stage	III	6 (5.8%)
	IV	99 (94.2%)
Type of chemotherapy	Carboplatin + paclitaxel	96 (91.4%)
	Carboplatin + docetaxel	2 (1.9%)
	Other	7 (6.7%)

ECOG PS: Eastern Cooperative Oncology Group performance status. TNM classification is shown depending upon the dates of patients' inclusion. TNM classification at those times were in accordance with the 7th or 8th edition.

**Table 2:** Relationships between laboratory parameters and ORR.

Parameter	Normal	Outside the norm	p-value
CRP	33 (86.8%)	36 (60.0%)	**0.006**
LDH	45 (75.0%)	24 (63.2%)	0.258
Albumin	63 (79.7%)	6 (31.6%)	**<0.001**
Sodium	59 (71.1%)	10 (66.7%)	0.763
Hemoglobin	54 (81.8%)	15 (46.9%)	**<0.001**
Neutrophils	50 (76.9%)	18 (56.3%)	0.058
NLR	39 (84.8%)	29 (56.9%)	**0.004**

CRP: C-reactive protein; LDH: lactate dehydrogenase; NLR: neutrophil/lymphocyte ratio

**Table 3:** Relationships between laboratory parameters and OS and PFS.

	Median PFS (95% CI), months			Median OS (95% CI), months		
Parameter	Normal	Outside norm	p-value	Normal	Outside norm	p-value
CRP	6.7 (5.8-8.5)	5.1 (3.3-6.5)	**0.001**	16.8 (12.9-NR)	11.7 (6.9-19.7)	**0.013**
LDH	6.0 (5.0-7.9)	5.2 (3.0-6.7)	0.083	19.7 (15.5-23.9)	9.6 (5.1-15.6)	**<0.001**
Albumin	6.5 (5.8-7.6)	1.3 (1.2-5.1)	**<0.001**	16.8 (12.9-21.2)	3.7 (1.6-NR)	**<0.001**
Sodium	6.0 (5.1-7.6)	4.8 (3.0-7.1)	0.055	16.3 (12.0-20.1)	6.3 (4.0-NR)	**0.007**
Hemoglobin	6.5 (5.7-7.8)	1.9 (1.3-6.0)	**0.023**	17.5 (12.9-22.1)	7.3 (3.4-NR)	**0.002**
Neutrophils	6.2 (5.3-7.8)	4.8 (1.7-7.0)	**0.040**	16.3 (12.1-21.2)	11.7 (4.1-NR)	**0.050**
NLR	6.9 (6.3-8.5)	4.0 (1.6-6.0)	**<0.001**	19.7 (16.3-NR)	9.4 (4.8-15.6)	**0.003**

CRP: C-reactive protein; LDH: lactate dehydrogenase; NR: not reached; NLR: neutrophil /lymphocyte ratio

**Table 4:** Multivariate Cox proportional hazards model for OS and PFS.

		OS		PFS	
Category	Subcategory	HR (95% CI)	p-value	HR (95% CI)	p-value
Gender	Male	Reference	x	Reference	x
	Female	1.428 (0.742; 2.748)	0.287	1.329 (0.799; 2.210)	0.273
Age		0.997 (0.967; 1.027)	0.828	0.996 (0.969; 1.024)	0.775
Smoking status	Non-smoker	Reference	x	Reference	x
	Ex-smoker	0.925 (0.398; 2.146)	0.856	0.825 (0.409; 1.664)	0.591
	Smoker	1.231 (0.580; 2.614)	0.588	0.929 (0.506; 1.707)	0.814
ECOG PS	0	Reference	x	Reference	x
	1	0.678 (0.211; 2.180)	0.515	0.293 (0.094; 0.912)	**0.034**
	2	1.134 (0.280; 4.596)	0.861	0.575 (0.152; 2.176)	0.415
CRP	Normal	Reference	x	Reference	x
	Outside norm	1.337 (0.728; 2.455)	0.349	1.886 (1.099; 3.237)	**0.021**
LDH	Normal	Reference	x	x	
	Outside norm	1.972 (1.074; 3.622)	**0.029**		
Albumin	Normal	Reference	x	x	x
	Outside norm	3.860 (1.845; 8.078)	**<0.001**	3.023 (1.681; 5.437)	**<0.001**
Sodium	Normal	Reference	x	x	
	Outside norm	1.980 (0.928; 4.228)	0.077		
Hemoglobin	Normal	Reference	x	Reference	x
	Outside norm	1.581 (0.836; 2.992)	0.159	1.282 (0.788; 2.088)	0.317
NLR	Normal	Reference	x	Reference	x
	Outside norm	1.818 (1.007; 3.281)	**0.047**	2.273 (1.427; 3.620)	**0.001**

CRP: C-reactive protein; ECOG PS: Eastern Cooperative Oncology Group performance status; LDH: lactate dehydrogenase; NLR: neutrophil/lymphocyte ratio

## Abbreviations

BEV: bevacizumab
CP: carboplatin and paclitaxel
CRC: colorectal cancer
CRP: C-reactive protein
CT: computed tomography
ECOG: Eastern Cooperative Oncology Group
ECOG PS: ECOG performance status
Hb: hemoglobin
LDH: lactate dehydrogenase
Neu: neutrophils
NLR: neutrophil/lymphocyte ratio
NSCLC: non-small cell lung cancer
ORR: overall response rate
OS: overall survival
PFS: progression-free survival
RECIST: Response Evaluation Criteria in Solid Tumors
VEGF: vascular endothelial growth factor
